# Field data on pre-season rice straw degradation using a microbial substrate and the effects on methane emissions during rice cultivation

**DOI:** 10.1016/j.dib.2023.109383

**Published:** 2023-07-06

**Authors:** Fauzi Jumat, Mohammad Hariz Abdul Rahman, Syuhaidah Abu Bakar, Nur Alyani Shakri, Rahiniza Kamaruzaman, Nurul Ain Abu Bakar, Mohd Aziz Rashid, Mohd Fairuz Md Suptian, Rashidah Ab Malek, Nur Liyana Zulkifle

**Affiliations:** a Agrobiodiversity & Environment Research Centre, MARDI, Serdang, Selangor 43400, Malaysia; b Paddy and Rice Research Centre, MARDI, Serdang, Selangor 43400, Malaysia

**Keywords:** Microbial substrate, Soil organic carbon, Soil nitrogen, Enzyme activity, Methane emission

## Abstract

Rice straw is one of the most abundant biomass wastes derived from rice cultivation activities. The current rice straw management practice during the wet (rainy) season in Malaysia involves the integration of straw into the soil. This practice offers both advantages and disadvantages to rice farmers and the environment. Straw integration may improve nutrient availability while concurrently causing high greenhouse gas (GHG) emissions due to the increase in soil carbon activity. In this work, the use of microbial substrate to enhance the degradation of straw was compared to an existing technique that used no additional inputs during soil integration. The data collected consisted of overall microbial enzyme production, soil organic carbon, soil nitrogen content, seasonal greenhouse gas emissions, plant characteristics, and crop yield. In brief, these data can be used as means of demonstrating the effects of improved straw degradation during the pre-season on the overall GHG emissions during the planting season.

**Specifications Table**

**Table:** 

Subject	Environmental chemistry
Specific subject areas	Microbial enzyme production, soil carbon content, soil nitrogen content, leaf physiological performance, crop yield, field methane (CH_4_) emissions
Type of data	Tables and figures
How the data were acquired	Nutrient characteristics of the substrate were outsourced to an accredited laboratory (MARDILab). Microbial enzyme production and enzyme activity: samples were collected from the field and determined using the dinitrosalicylic acid (DNS) assay, cellulase assay, xylanase assay, and laccase assay. Soil organic carbon: samples were collected from the field and determined using a standard titration method. Soil nitrogen content: samples were collected from the field and determined using the Kjeldahl method. Leaf physiological performance: assessment of net photosynthetic rates, stomata conductance, transpiration rate, and chlorophyll fluorescence. Leaf chlorophyll content: destructive and non-destructive chlorophyll determination. Crop yield: determined using a crop-cutting test (CCT) in a 1 m × 1 m area. CH_4_ fluxes: calculated from gas samples collected using the static chamber method from the field and analysed using gas chromatography.
Data format	Raw and analysed data
Description of data collection	The straw was integrated into the soil after a week of microbial substrate addition (first soil tillage process), and only two straw sampling sessions could be carried out. In a concurrent process, the soil samples used to analyse the soil microbial enzyme activity, soil organic carbon, and soil nitrogen content were taken every 14 days, beginning from the microbial substrate application. The soil samples were taken at a depth of 0–20 cm, with two (2) types of samples taken to determine (a) soil microbial enzyme activity and (b) soil nutrient content. The samples were analysed from the pre-planting season (54 days before seeding). This analysis continued during the planting season until harvesting (98 days after seeding). To determine the enzyme activity, the soil samples were mixed with distilled water and shaken homogenously before the analysis. For the soil nutrient analyses, the soil samples were first dried in open air for an average of seven days before the analyses. Data for the leaf physiological performance and chlorophyll content were analysed at three different stages of plant growth (tillering, flowering, and ripening), while the crop yield was determined using a CCT during harvesting. Meanwhile, gas flux measurements were taken from eight days after seeding (DAS) until 98 DAS. All data were saved in XLSX data files.
Data source location	Sungai Besar, Selangor, Malaysia (latitude: 3.729782, longitude: 101.031646)
Data accessibility	Repository name: Mendeley Data Data identification number: 10.17632/xk4sdppkwy.3 Direct URL to data: https://data.mendeley.com/datasets/xk4sdppkwy/3

## Value of the Data

• The data describe the effects of microbial substrate application to improve straw decomposition, with the substrate having been integrated into the soil on the field during the pre-planting season.
• Improved straw decomposition in the integrated soil was associated with a drop in soil organic carbon, which contributed to an overall reduction in methane (CH_4_) emissions during rice cultivation.
• Using the microbial substrate will potentially benefit farmers by improving soil and plant nutrient characteristics while simultaneously reducing greenhouse gas (GHG) emissions.
• The data can be re-used to compare the trend of soil microbial activity and nutrient characteristics in relation to methane (CH_4_) emissions at various locations of rice cultivation for future studies.

## 1 Objective

The effects of microbial enhancement on rice straw degradation were rarely observed holistically using different set of parameters especially those involving a field scale trial. Therefore, this study was conducted to evaluate these effects based on the changes in soil nutrient characteristics, soil enzyme activities, plant physiological performances, and overall field methane (CH_4_) emissions.

## 2 Data Description

The data in this study included raw, descriptive (means), and analysed data for one season of the rice cultivation cycle undertaken in Sungai Besar, Selangor, Malaysia. The area studied was within the northwestern region of Integrated Agriculture Development Area (IADA), one of the main rice granary areas in Malaysia. The data were organised into four tables and seven figures to describe the characteristics of the microbial substrate used for the application on rice straw, as well as the soil enzyme activity, soil nutrient characteristics, plant physiological characteristics, and methane (CH_4_) fluxes assessment conducted throughout the rice-cropping season. The study was conducted to potentially find an alternative option for rice straw management in the field, as well as determine the possibility of conducting similar studies. Damaged paddy grains were used as the microorganism substrate mainly because they were rice mill leftovers with no current economic value, although they had certain beneficial nutrient characteristics. Table 1 shows the nutrient comparison between the rice husk and the damaged paddy grains. The table indicates that certain additional nutrients were available in the damaged paddy grains compared to the rice husk.

**Table 1:** Comparison of the characteristics of rice husk and damaged paddy grains (substrate).

Type	Rice husk	Damaged paddy grain (substrate)
Lignin (%)	21.00 ± 0.68	17.87 ± 2.54
Hemicellulose (%)	16.31 ± 0.43	16.30 ± 0.71
Cellulose (%)	43.62 ± 1.84	41.74 ± 2.57
Starch (%)	9.67 ± 1.15	10.29 ± 0.94
Total Sugar (%)	0.23 ± 0.08	1.35 ± 0.00
Reducing Sugar (%)	0.00	0.00
Crude Protein (%)	3.42 ± 0.54	6.09 ± 0.14
Carbon (%)	28.12 ± 0.37	29.21 ± 0.45
Nitrogen (%)	0.43 ± 0.05	0.94 ± 0.10

**Fig. 1:**
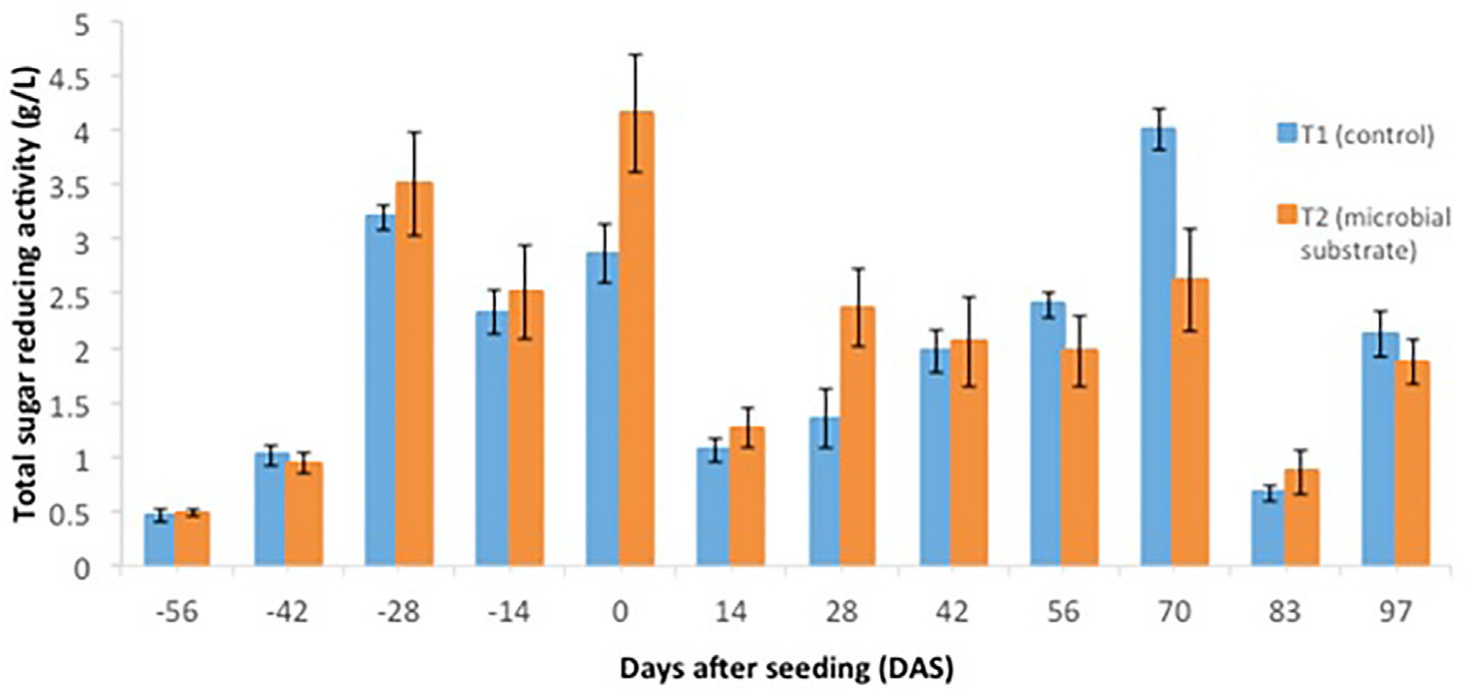
Total sugar-reducing activity between control and treatment of microbial substrate throughout the season.

**Fig. 2:**
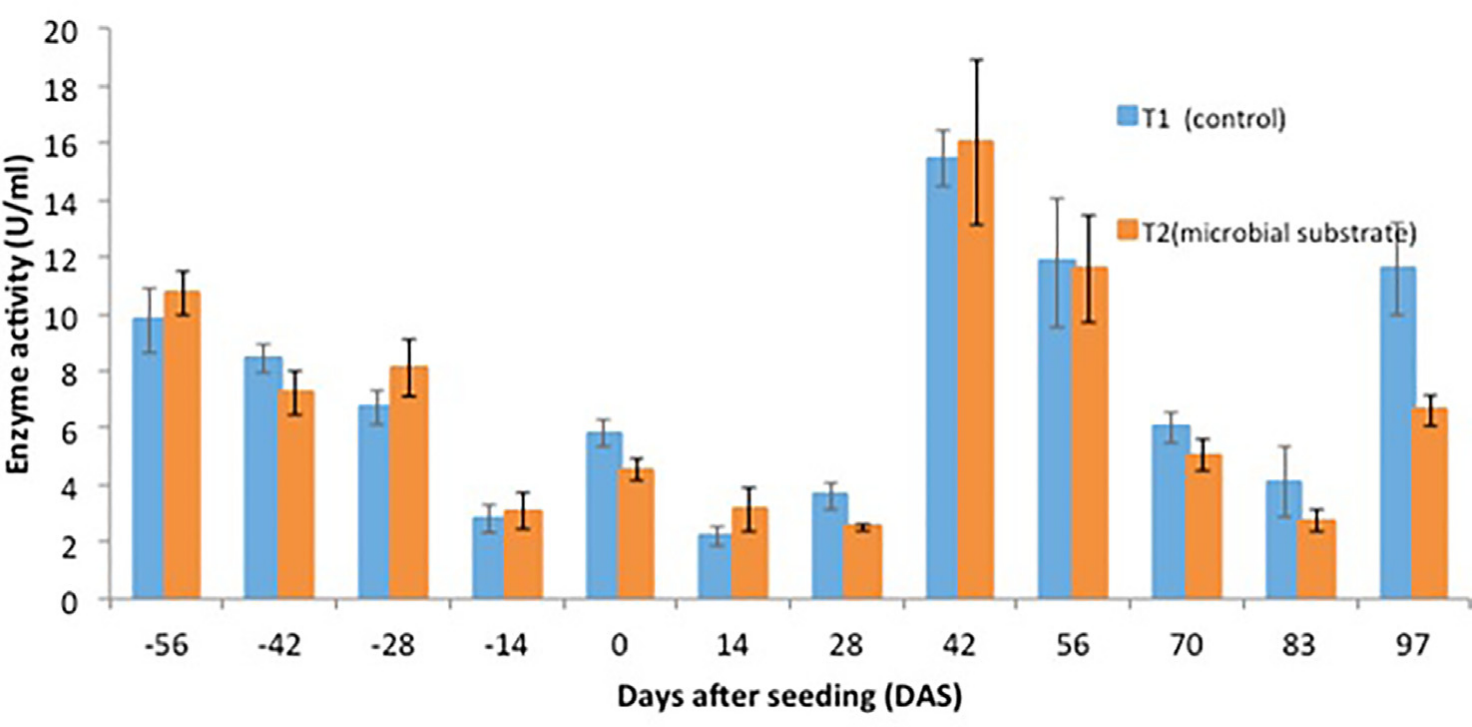
Cellulase enzyme activity between control and treatment of microbial substrate throughout the season.

**Fig. 3:**
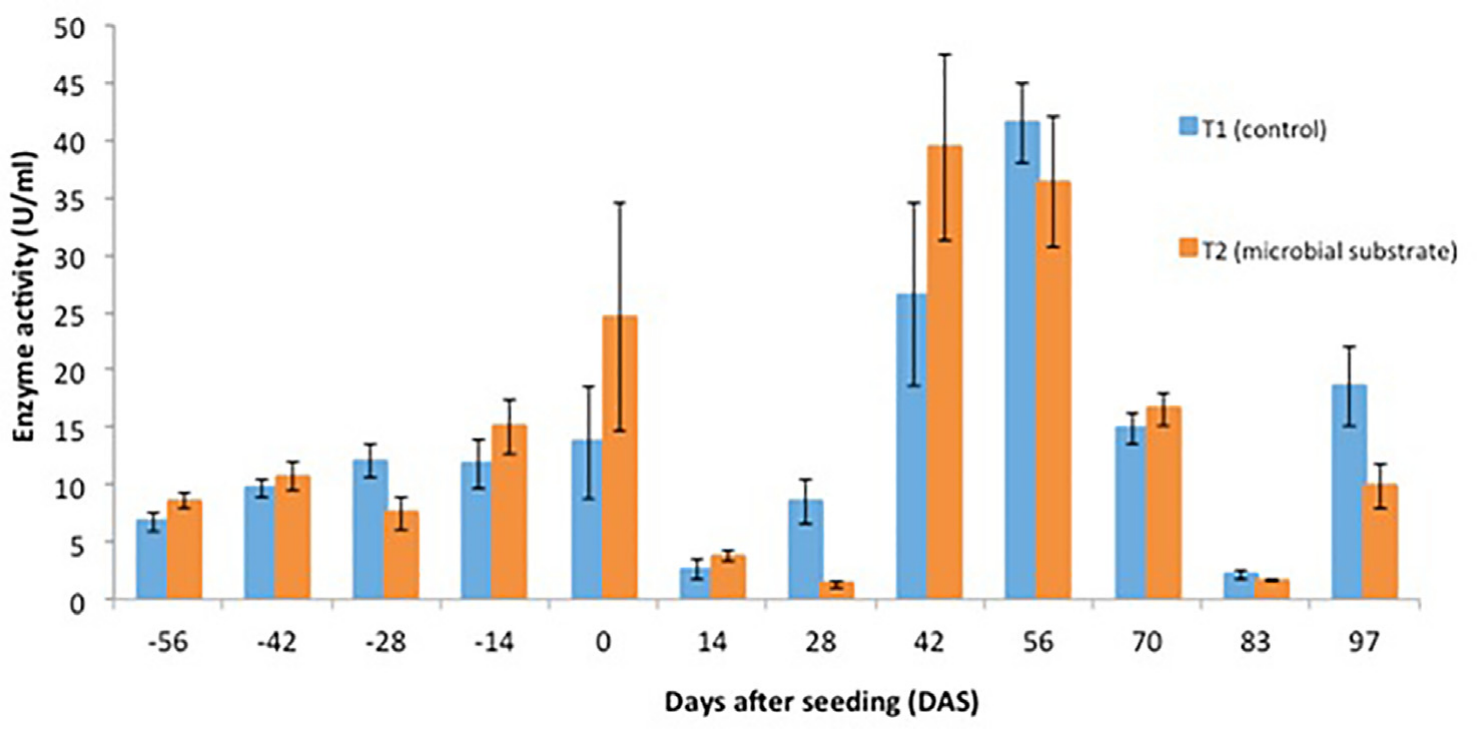
Xylanase enzyme activity between control and treatment of microbial substrate throughout the season.

**Fig. 4:**
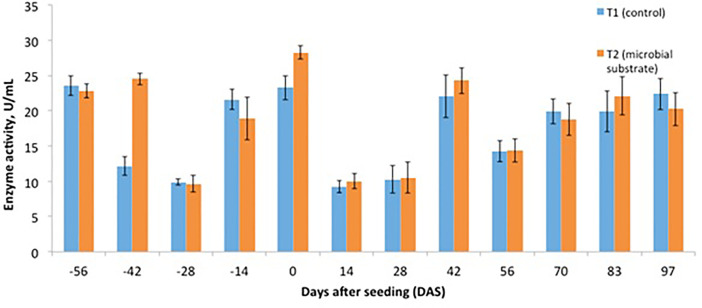
Laccase enzyme activity between control and treatment of microbial substrate throughout the season.

**Fig. 5:**
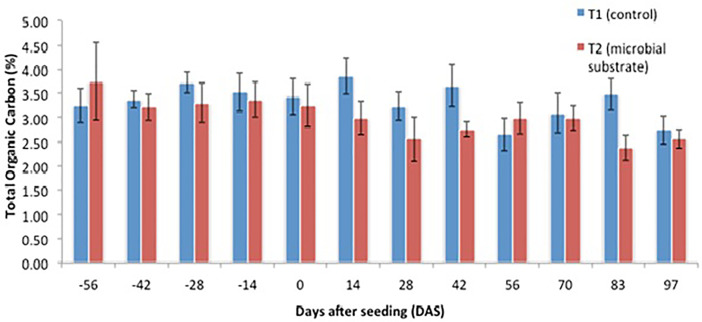
Soil organic carbon between control and treatment of microbial substrate throughout the season.

**Fig. 6:**
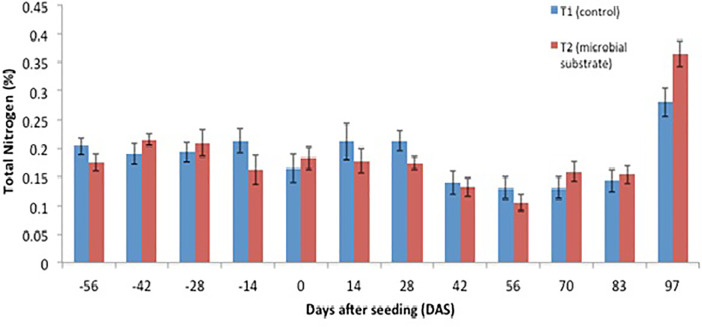
Soil nitrogen content between control and treatment of microbial substrate throughout the season.

**Fig. 7:**
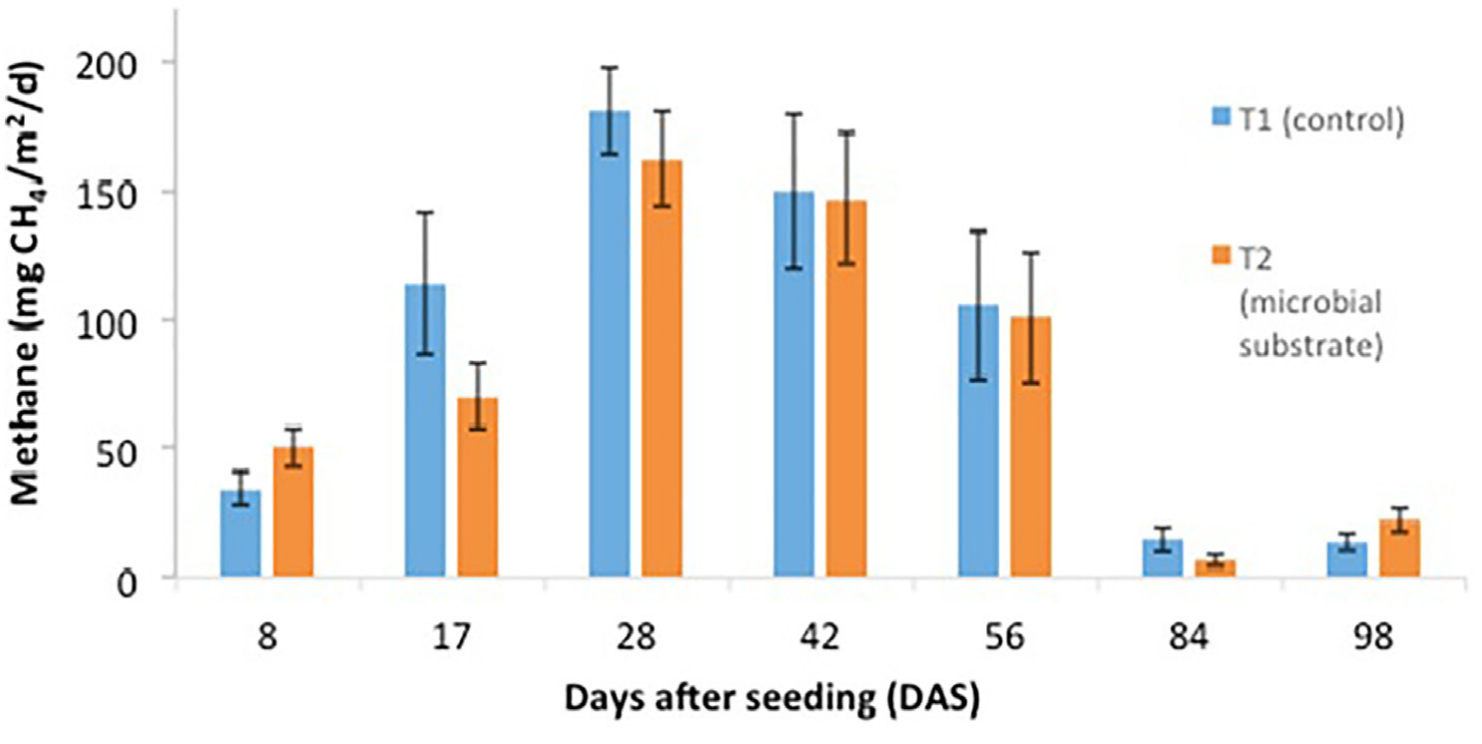
Comparison of methane (CH_4_) emissions between control and treatment of microbial substrate throughout the season.

Other data are presented, including (1) soil microbial enzyme activity, including a dinitrosalicylic acid (DNS) assay (Fig. 1, Fig. 2, Fig. 3, Fig. 4), (2) soil organic carbon changes (Fig. 5), (3) soil nitrogen changes (Fig. 6), and (4) seasonal methane (CH_4_) emissions (Fig. 7). The soil microbial enzyme activity, organic carbon changes, and nitrogen changes were evaluated from pre-planting (beginning at 56 days before seeding or -56 days after seeding, DAS) and throughout the planting season until immediately before harvesting at 97 DAS. The raw data for these analyses were kept in the data file called “Enzyme activity data.xlsx,” “Carbon contents.xlsx,” and “Nitrogen contents.xlsx” in the Mendeley repository. Meanwhile, the total methane (CH_4_) emissions were measured during the early planting stage (seeding) until 98 DAS, at which time the plants were still under anaerobic conditions. Water was allowed to flow out of the field one to two days after the last gas sampling to prepare for harvesting. The raw data collected for the gas emissions were kept in the data file called “Methane emission data.xlsx” in the Mendeley repository. Plant physiological performance was analysed at three stages of planting: tillering, flowering, and ripening (Tables 2 and 3). The data were kept in data file called “Plant physiology and chlorophyll.xlsx.” A summary of the data is given in Table 4. The data showed a (significant) reduction in soil organic carbon content with the use of microbial substrate. The results also revealed an increased enzyme activity from sugar reducing, resulting in high levels of DNS values, xylanase, and laccase enzyme activities. Concurrently, methane (CH_4_) emissions reduced during the rice cultivation season. In this study, the microbial substrate comprised a combination of three microbial fungi from *Aspergillus* and *Trichoderma* genera, which were initially isolated from the rice field. These microbes included fungi coded as AMF1 and AMF2, while the third fungus was coded as Trichoderma 1614*.* However, due to various regulatory issues, it is suggested that future rice straw degradation trials to solely use Trichoderma 1614.

**Table 2:** Leaf physiological performance for treatments at different growth stages.

	Treatment	Net photosynthetic rate (A) µmol/m^2^/s	Stomatal conductance (gs) mol/m^2^/s	Leaf transpiration rate (E) mmol/m^2^/s	Chlorophyll fluorescence (Fv/Fm ratio)
Tillering stage	T1 (control)	18.51 a	1.64 a	7.32 a	0.50 a
	T2 (microbial substrate)	18.21 a	1.62 a	7.40 a	0.54 a
Flowering stage	T1 (control)	19.22 a	1.18 a	7.72 a	0.76 a
	T2 (microbial substrate)	16.42 a	1.32 a	7.62 a	0.80 a
Ripening stage	T1 (control)	18.30 a	0.75 a	6.72 b	0.72 a
	T2 (microbial substrate)	18.23 a	0.79 a	7.22 a	0.73 a

**Table 3:** Leaf chlorophyll content for treatments at different growth stages.

	Treatment	Chlorophyll A (mg/cm^2^)	Chlorophyll B (mg/cm^2^)	Total chlorophyll (mg/cm^2^)	Relative chlorophyll content (SPAD)
Tillering stage	T1 (control)	4.10 a	1.21 a	5.31 a	31.25 a
	T2 (microbial substrate)	5.13 a	1.69 a	6.81 a	32.58 a
Flowering stage	T1 (control)	4.87 a	2.51 a	7.38 a	34.06 a
	T2 (microbial substrate)	4.99 a	2.58 a	7.57 a	32.84 a
Ripening stage	T1 (control)	1.87 b	0.63 a	2.50 b	27.53 b
	T2 (microbial substrate)	2.41 a	0.87 a	3.28 a	32.24 a

**Table 4:** Comparison of the mean values between treatments.

Treatments	T1 (control)	T2 (microbial substrate)	% difference (T2>T1)
Straw organic C% (Day 1)	30	29.6	-
Straw organic C% (Day 7)	31.7	30	-
Sugar-reducing activity (g/L)	1.97a	2.05a	+4.06
Cellulase (U/mL)	7.42a	6.78a	−8.63
Xylanase (U/mL)	14.18a	14.61a	+3.03
Laccase (U/mL)	17.53a	18.70a	+6.67
Soil organic C%	3.33a	3.04b	−8.71
Soil N%	0.19a	0.18a	−5.26
Emission factor (kg/ha/d)⁎	0.92a	0.82a	-
Cumulative CH_4_ emission (kg/ha)	82.42	74.06	−10.14
Yield (kg/ha)	6768.78a	6976.11a	+3.06

Means followed by the same letters within a column are not significantly different at (P>0.05).

⁎ Statistical means were calculated separately for emission factor.

## 3 Experimental Design, Materials and Methods

### 3.1 Material For Microbial Substrate

The substrate used in this experiment was formed from damaged paddy grains obtained from Sungai Burong Rice Mill in Sungai Besar, Selangor, Malaysia. These grains were rice-processing by-products and constituted approximately 5% of the overall processed products/ grains. The obtained substrate (grain) was first dried at room temperature and subsequently ground into powder using a hammer mill (TECO brand, model FFC37). The powdered substrate was then kept at room temperature before analysis and further mixture with the selected microbes.

In the next step, the powdered substrate was autoclaved at 121°C for 15 min for eventual use in the microbial mixing stage. To prepare the microbes, the fungi were inoculated in potato dextrose agar for one week in an incubator at 27°C. After seven days, the stock spore was prepared using sterile 0.1% Tween 80. Then, 5 mL of 0.1% Tween 80 was added to the fungi plate. Next, all the contents were scraped from the petri dish. Following this, the solution was extracted into 25 mL of 0.1% Tween 80 in a Schott bottle for the spores to be counted to the desired concentration using a hemacytometer method. In this experiment, the spore cell concentration used was 1 × 10^7^. Next, the calculated cultured stock was diluted in 8 L of 0.1% Tween 80, after which the solution was poured into 80 kg of the autoclaved powdered substrate and mixed with 5% of 4 L of brown sugar solution. Finally, the components were mixed and left to rest for seven days to allow the microbes to grow and multiply before application on the field.

### 3.2 Experimental Design and Preparation

The experiment was conducted between February 2019 and July 2019 on two (2) 0.6-hectare rice cultivation plots. The experimental plots were subject to the following treatments: (1) non-added microorganism substrate (control) and (2) microbial substrate degrader application at 80 kg/lot. In this experiment, since each treatment plot measured 0.6 ha (or 0.5 lot as one lot is equivalent to 1.2 ha), the substrate was applied at 40 kg per designated plot (treatment 2). Meanwhile, the rice variety used in the study was MR 219, and the cultivation method was direct seeding. The cultivation technique followed standard agronomic rice cultivation practices and standard water management involving continuous flooding.

The three soil tillage processes were applied before the planting season began. The first tillage was carried out a week after microbial substrate application. The second tillage was carried out two weeks before rice planting (seeding), and the third tillage was carried out one week before rice planting. Overall, three fertilisation stages were applied. *NPK Sebatian* (17.5% N, 15.5% P, 10% K) was applied at 15 DAS, urea (46% N) at 35 DAS, and *NPK Tambahan* (17% N, 3% P, 25% K, 2% MgO) at 55 DAS. Additionally, there were also records of specific organic amendments using products from the brand Deliver™ being applied before the planting season. However, these were not applied during the experimental season. Similarly, the pesticides were also applied following standard subsidised input methods.

### 3.3 Data Collection and Analysis

#### 3.3.1 Characterisation of Damaged Paddy Grains As A Microbial Substrate

The powdered substrate samples (triplicate) were taken for nutrient analyses namely lignin (%), cellulose (%), hemicellulose (%), starch (%), total sugar (%), reducing sugar (%), crude protein (%), carbon (%), and nitrogen (%). The samples were sent to a commercial and accredited laboratory, MARDI Laboratory (MARDILab) at Serdang, Selangor, Malaysia for analysis. However, the organic C and N analyses were conducted in-house using the standard method of [1,2]. The data obtained from the analysis were comparable to the study in [3].

#### 3.3.2 Collection of Soil Samples

Soil samples for the organic carbon and nitrogen analysis were collected at a depth of 0–20 cm using a manual auger at nine sampling points for each treatment. All the soil samples were taken after the gas sampling process if the sampling was conducted on the same date. Following this, the samples collected were kept in a chiller (7°C) for a maximum of 24 h upon their transfer to the laboratory. In order to conduct the soil nutrient analyses, the samples were air-dried for seven days and subsequently sieved using a 150-micron nylon sieve. The soil samples were then ground using the laboratory's mill (model PSY MP 20) with a 0.8 sieve capacity. Meanwhile, the soils were directly extracted for further test upon chilling if they were to be analysed for soil enzyme activity.

#### 3.3.3 Evaluation of Soil Properties Including Enzyme Activity

The ground soil samples were analysed for organic carbon (C) and nitrogen (N) characteristics using standard laboratory methods. Organic Carbon (OC) was analysed using the method outlined by [1]. Using this method, the samples were oxidised by potassium dichromate (K_2_Cr_2_O_7_) with the addition of sulphuric acid (H_2_SO_4_) before being titrated with ammonium (II) sulphate [(NH_4_)_2_Fe(SO_4_)_2_(H_2_O)_6_], with the results presented in percentages of OC. Specifically, soil organic carbon is associated with plant residues, and mixing rice straw into soil will rapidly increase the organic carbon before it gradually decreases through decomposition [4]. Thus, a trend of effective straw decomposition in the soil was observed through the reduced percentage of soil OC in the field. Additionally, straw is expected to degrade better when integrated into the soil partly due to maintained soil moisture content, which supports microbial decomposition activities. Meanwhile, the total N was determined using the Kjeldahl method [2], whereby the samples were digested with sulphuric acid (H_2_SO_4_), followed by distillation with sodium hydroxide (NaOH) and titration with hydrochloric acid (HCl) to obtain the N percentage.

For all the soil enzyme assays, the collected soil samples were initially weighed (5 g), mixed with 45 mL of distilled water, and shaken homogenously. For the sugar-reducing assay, 1 mL of soil samples was added to 3 mL of DNS in test tubes. Three drops of sodium hydroxide (NaOH) were then added to the solution. For the blank analysis, the sample was substituted with distilled water. The samples were then boiled in 100°C water for five minutes, and colour changes were observed. After five minutes, the samples were put in room-temperature water, and 20 mL of distilled water was added to the samples. The samples were read using a UV spectrometer at 540 nm.

For the cellulase enzyme assay, 1 mL of 0.1 M citrate buffer, 1 mL of 0.5% carboxymethylcellulose (CMC), and 1 mL of the samples were added to the test tubes. The samples were then incubated in a water bath at 30°C for 30 min. Following this, 3 mL of DNS was added to the samples and mixed thoroughly before continued boiling in 100°C water for three minutes. After that, the samples were put in room-temperature water, and 20 mL of distilled water was added to the samples. The samples were read using a UV spectrometer at 540 nm. The steps for the xylanase enzyme assay were similar to those in the cellulase enzyme assay. The only difference was substituting 0.5% CMC with 0.1% xylanase.

For the laccase enzyme activity assay, 1 g of soil was suspended in 9 mL of distilled water, and the extract samples were filtered using filter paper. Then, 1 mL of each sample was added into a test tube with 1 mL of 10 mM sodium acetate and 200 µl of 0.2 mM 2,2’-azino-bis(3-ethylbenzothiazoline-6-sulfonic acid (ABTS). The mixture was then incubated at room temperature for 15 min before the samples were read using a UV-spectrometer at 420 nm.

During the enzyme analyses, there was one missing data at one of the replicates, which happened at T1 (83 DAS). Thus, the average of the datasets comprised eight instead of the usual nine points (replicates).

#### 3.3.4 Evaluation of Leaf Physiological Performance, Leaf Chlorophyll Content and Crop Yield

The plant leaf physiological performance and chlorophyll content were analysed at three phases of growth (tillering, flowering, and ripening). The net photosynthetic, stomata conductance, and transpiration rates were determined using a portable photosynthesis system (LI-6400XT, LICOR Inc., Nebraska, USA). The chlorophyll fluorescence was measured using a portable plant efficiency analyser (PEA) (FMS 2, Hansatech Instruments Ltd., UK), with the ratio of Fv/ Fm used to determine the leaf chlorophyll fluorescence.

The relative chlorophyll content of the rice leaves was determined using a portable chlorophyll SPAD meter (SPAD-502, Konica Minolta, Japan). Meanwhile, the destructive chlorophyll determination was carried out by following a chlorophyll extraction technique using acetone. These steps were followed by absorbance reading at 664 nm and 647 nm wavelengths of the chlorophyll extracts using a spectrophotometer. The a, b, and total chlorophyll content were calculated using the equations proposed by [5].

The grain yield analysis was carried out using a crop-cutting test (CCT) using an area dimension of 1 m × 1 m. First, the harvested grain was dried, winnowed, and weighted. Following this, the estimated weight was measured by converting the measured area into per unit area (kg/ha), based on the 14% grain moisture content. During the cultivation season, the harvest date for the field occurred at 105 DAS.

#### 3.3.5 Measurement of Methane (CH_4_) Emissions

The methane (CH_4_) was measured using a static chamber method [6]. Daily methane (CH_4_) samples were taken at six points on each treatment plot where scaffolding or boardwalks had been built around the sampling points. The sampling points were established approximately two metres from the non-submerged land (locally called *batas*). This approach was taken to ensure no soil disturbances that might cause artificial methane (CH_4_) ebullition would occur while the sampling was being carried out. Specifically, the chamber dimensions were measured 110 cm in height and 35 cm in width and length (110 cm × 35 cm × 35 cm). Gas samples were collected from the field using 20 mL syringes with hypodermic needles. Daily flux methane emission samples were collected at 10-min intervals from 9 a.m. until 9.30 a.m. at 0, 10, 20, and 30 min. Meanwhile, gas samples were collected at two-week intervals, beginning eight DAS and 17 DAS, 28 DAS, 42 DAS, 56 DAS, 84 DAS, and 98 DAS. The final samples were collected immediately before the water was drained from the field before harvesting. The gas samples were analysed using gas chromatography (SRI 8610C).

The daily methane flux was calculated using the following formula:

$$CH_{4\text{flux}}=\frac{\Delta C}{\Delta t}\;\times \frac{V}{A}\;\times \rho \;\times \frac{273}{273+T}$$

where Δ*C / Δt* is the change in concentration in parts per million (ppm) over time; V is the volume of the chamber in m^3^; A is the chamber area in m^2^; ρ is the gas density (0.717 kg m^−3^); and T is the air temperature in the chamber in°C.

Meanwhile, the total methane (CH_4_) emissions were calculated using an integration method. This was similar to the method highlighted in [7] and used the following formula:

$$\text{Total}CH_{4\text{flux}}=\sum_{i=1}^{n-1}\left(R_{i}xD_{i}\right)$$

where n is the number of sampling intervals; $R_{i}\;$is the mean rate of CH_4_ flux (mg m^−2^ d^−1^) within the two sampling intervals; and $D_{i}$ is the number of days within the sampling interval.

Due to technical issues, sampling was not possible at 70 DAS, and the average of the values between 56 DAS and 84 DAS was treated as the total emissions. There were also missing data at one of the six points (replicates), which happened at T1 (8 DAS) and T2 (17 DAS). Thus, the average of these two datasets comprised five instead of the usual six points (replicates).

#### 3.3.6 Data Analysis

Analysis of variance (ANOVA) was carried out to determine the statistical model for the main factors. Additionally, the means were analysed for significance using least significant difference (LSD) tests to analyse the plants, while the remaining data were analysed using a Duncan's test. All the analyses were carried out using the statistical analysis system (SAS) version 9.3 (SAS Institute, Inc., USA).

## Ethics Statements

Not applicable. This work did not involve any types of animal or human studies.

## CRediT authorship contribution statement

**Fauzi Jumat:** Conceptualization, Methodology, Formal analysis, Writing – original draft, Project administration, Funding acquisition. **Mohammad Hariz Abdul Rahman:** Methodology, Writing – review & editing, Formal analysis. **Syuhaidah Abu Bakar:** Methodology, Formal analysis. **Nur Alyani Shakri:** Methodology, Formal analysis. **Rahiniza Kamaruzaman:** Visualization, Investigation. **Nurul Ain Abu Bakar:** Methodology, Formal analysis. **Mohd Aziz Rashid:** Formal analysis, Writing – review & editing, Validation. **Mohd Fairuz Md Suptian:** Validation. **Rashidah Ab Malek:** Methodology, Formal analysis. **Nur Liyana Zulkifle:** Methodology, Formal analysis.

## Declaration of Competing Interest

The authors declare that they have no known personal relationships or competing financial interests that may have influenced the work reported in this article.

## Data Availability

Field study on the effects of microbial substrate application on rice straw degradation (Original data) (Mendeley Data).
